# Una historia de la eugenesia en el Chile del siglo XX: en el nombre del padre, de la ciencia y de la raza

**DOI:** 10.1590/S0104-59702023000100012

**Published:** 2023-04-03

**Authors:** Felipe Martínez-Fernández

**Affiliations:** i Postgraduate Researcher, School of Philosophy, Religion and History of Science/University of Leeds. Leeds – West Yorkshire – UK. fel.martinezf@gmail.com

Las trayectorias biográficas del ex dictador militar Augusto Pinochet y el ex presidente socialista Salvador Allende parecen tener puntos de encuentro. Ambos, nacidos en las primeras décadas del siglo XX, fueron testigos de una serie de cambios culturales, políticos y sociales en la historia contemporánea de Chile. Pinochet y Allende realizaron sus estudios secundarios en prestigiosos colegios en la ciudad de Valparaíso, y ambos compartieron valores sociales que emergieron como ejes claves del bienestar del país: la familia, los vínculos matrimoniales y el compromiso público por la nación.

Allende, quién realizó su servicio militar en el Regimiento Coraceros a mediados de la década de 1920, y que finalizó sus estudios de medicina en la Universidad de Chile en 1932, lideraría importantes reformas en salud pública hacia 1939. En particular, la eugenesia emergió en sus propuestas como una herramienta científica, y que proveía soluciones de vanguardia para los cambios y demandas sociales (Illanes, 1993). Pinochet continuaría sus estudios en la Academia de Guerra hacia finales de la década de 1940, especializándose en geografía militar y geopolítica. Los conocimientos científicos procedentes de ambas especialidades fueron decisivos durante la dictadura chilena, cuyas políticas de urbanismo impusieron desplazamientos forzados, la creación de asentamientos periféricos y la segregación de los sectores populares (Leyton Robinson, 2020).

Las historias cruzadas de Allende y Pinochet convergen en las reflexiones finales de *The religion of life*, una historia de la eugenesia en Chile, y escrito por la historiadora norteamericana Sara Walsh (2022). La autora delinea los contornos de una ciencia que pareció, en casos concretos y específicos, amplificar las jerarquías sociales y de género, además de dar cuenta del nuevo estatus social de la ciencia. Se utilizan fuentes impresas, entre las que se incluyen revistas médicas, trabajos científicos, prensa, y publicaciones del ámbito intelectual católico. Estas últimas constituyen una novedad historiográfica y permiten hacer un contrapunto a los discursos médicos.

Su novedad metodológica radica en dos aspectos. Por un lado, la autora propone releer la eugenesia en clave colonial, una revisión a los procesos invisibilizados del mestizaje colonial, en los que los usos y las interpretaciones de las ciencias biomédicas amplificaron las inequidades étnicas y raciales. Por otro lado, da cuenta de las dimensiones de género y de cómo los discursos científicos y religiosos escenificaron las asimetrías de poder entre lo masculino y lo femenino.

*The religion of life* está dividido en dos partes. En los primeros tres capítulos, la autora explora los vínculos conceptuales y teóricos entre el catolicismo y la eugenesia. En el primer capítulo, la autora sitúa las primeras convergencias católicas y eugenésicas a través de la protección y extensión del matrimonio. La crisis matrimonial – y sus consecuencias legales y morales – supuso no solo una pérdida de los valores reproductivos y familiares, sino que también implicó la protección de la integridad espiritual y biológica del país. La coexistencia eugenésica entre instituciones del Estado y la Iglesia Católica fue posible en un contexto en donde la extensión de la vida se consagró desde un doble estatus, médico y sacerdotal. El segundo capítulo da cuenta de las distintas interpretaciones sobre el origen de la existencia humana. La creciente influencia de los conocimientos científicos en el ámbito de la biología entra en contradicción con valores que hasta entonces habían sido predominio de la fe católica y el misterio divino. Sin embargo, tanto la teoría científica como la doctrina católica convergieron en una serie de aspectos, como la observación, la contemplación y el predominio de Dios dentro de la jerarquía de la creación humana. El tercer capítulo retoma las clásicas interpretaciones eugenésicas en Latinoamérica en torno a una mayor influencia del medioambiente por sobre los determinismos biológicos (Stephan, 1991). Las altas tasas de alcoholismo, tuberculosis y mortalidad infantil ampliaron los márgenes coercitivos del poder médico, particularmente para los casos de esterilización forzada. A juicio de la autora, fueron voces católicas las que modelaron la acción eugenésica, estableciendo no solo límites éticos a la investigación médica, sino que también límites a los nacientes estados de bienestar.

En la segunda parte del libro, la autora analiza los discursos raciales e imaginarios de género. En el cuarto capítulo, las conmemoraciones del primer centenario de la República, en 1910, permitieron que distintas voces resignificaran la excepcionalidad, virilidad y homogeneidad de la llamada “raza chilena”. La masculinización de la sociedad convivió, a su vez, ante los continuos miedos y fobias de mezclas raciales, lugar en donde las pigmentaciones oscuras de la piel podían revelar un retroceso civilizatorio. El quinto capítulo examina los imaginarios de futuro y la reproducción familiar. El ideario eugenésico se orientó a las preocupaciones poblacionales bajo la lupa de la maternidad como una obligación patriótica. Asimismo, la autora recuerda que las pretensiones de maternidad fueron leídas en clave colonial, en donde mujeres indígenas parecieron no tener cabida en los relatos del mestizaje. Finalmente, el capítulo sexto da cuenta de las formas más visibles de las distinciones eugenésicas. Postales, tarjetas y revistas evidenciaron que los rostros de la pobreza podían ser visiblemente reconocidos en pieles oscuras. A su vez, la importancia médica por documentar visualmente estados que parecían ser patológicos, como la intersexualidad, tuvo como contraparte una exposición pública de cuerpos, en las que malformaciones congénitas y su exposición a comunidades científica amplificaban las asimetrías raciales, sexuales y de género.

*The religion of life* es un libro importante y que establece cruces teóricos y metodológicos entre la historia cultural de la ciencia y los estudios de género. Se suma además a una literatura que ha comenzado a estudiar los vínculos entre eugenesia y la religión católica, en el contexto de culturas latinas (Cleminson, 2014). Ofrece evidencias históricas que visibilizan los efectos aún disonantes del periodo colonial latinoamericano en pleno siglo XX. La portada del libro asimila la cartografía geográfica a una cartografía genética. Un mosaico de diversos colores, múltiples en fragmentos, tonalidades y pigmentos, y que de vez en cuando parecen ser olvidados en las historias contemporáneas de Chile.
